# DeepCAC: a deep learning approach on DNA transcription factors classification based on multi-head self-attention and concatenate convolutional neural network

**DOI:** 10.1186/s12859-023-05469-9

**Published:** 2023-09-18

**Authors:** Jidong Zhang, Bo Liu, Jiahui Wu, Zhihan Wang, Jianqiang Li

**Affiliations:** 1 Faculty of Information Technology, Beijing University of Technology, Beijing, 100124 China; 2 School of Mathematical and Computational Sciences, Massey University, Auckland, 0745 New Zealand

**Keywords:** Bioinformatics, Attention mechanism, DNA transcription factors sequence, Convolutional neural networks

## Abstract

Understanding gene expression processes necessitates the accurate classification and identification of transcription factors, which is supported by high-throughput sequencing technologies. However, these techniques suffer from inherent limitations such as time consumption and high costs. To address these challenges, the field of bioinformatics has increasingly turned to deep learning technologies for analyzing gene sequences. Nevertheless, the pursuit of improved experimental results has led to the inclusion of numerous complex analysis function modules, resulting in models with a growing number of parameters. To overcome these limitations, it is proposed a novel approach for analyzing DNA transcription factor sequences, which is named as DeepCAC. This method leverages deep convolutional neural networks with a multi-head self-attention mechanism. By employing convolutional neural networks, it can effectively capture local hidden features in the sequences. Simultaneously, the multi-head self-attention mechanism enhances the identification of hidden features with long-distant dependencies. This approach reduces the overall number of parameters in the model while harnessing the computational power of sequence data from multi-head self-attention. Through training with labeled data, experiments demonstrate that this approach significantly improves performance while requiring fewer parameters compared to existing methods. Additionally, the effectiveness of our approach  is validated in accurately predicting DNA transcription factor sequences.

**Supplementary Information:**

The online version contains supplementary material available at 10.1186/s12859-023-05469-9.

## Introduction

Most of the known genetic variants of human diseases are often closely related to human genes [1, 2]. These genetic variants also generally contain information that is mostly hidden in certain regions of the genome. Therefore, it is particularly important to discover the functional locations of the genome in order to gain a broader understanding of how genes work. The genome contains two regions, open and closed, and most of the transcriptional processes occur in the open region. Genes, the basic unit of genetics, are special segments of DNA that have genetic utility, while DNA is the important participant in biological processes such as splicing, translation and transcription [3, 4]. The effective identification and recognition of functional and genetic properties in DNA can be achieved by relying on traditional biological experiments, especially in the study of DNA transcription factors. Transcription factors are important molecules that control gene expression and directly control the timing and extent of gene expression. Gene expression is regulated by the activation or repression of transcription factors, which are essential for a number of critical cellular processes. However, these biological experiments are expensive and time-consuming in the face of large-scale classification tasks, which often become labor-intensive to obtain complete results [5, 6]. The drawbacks of these biological experiments that continue to facilitate the development of computational methods for uncovering the genetic information contained in DNA transcription factors sequences. These achievements have not only contributed to the development of biology itself, but also enabled the advancement of the studies surrounding biology, such as cancer [7].

Recently, research has increasingly focused on the challenges of properly predicting the functionality or properties of sequences in traditional biological experiments, such as DNA transcription factors. With the recent development of high-throughput technologies, different sequencing technologies such as MNase-seq [8, 9] and ATAC-seq [10] have been generated to fit different research purposes, and these technologies have led to a significant enrichment of relevant sequence datasets. The sophistication of these data makes it very difficult to accurately predict the functionality or properties of sequences by conventional biological experiments. Precisely because of this, progressively some computational or machine learning based methods started to be applied to sequence analysis. As an extensive DNA transcription factor analysis tool, MEME analyzes DNA sequences by building a maximum expectation model [11]. A method based on k-mer with SVM [12] called gkm-SVM [13] achieves better performance than the classical k-mer SVM for classification in the ENCODE Chip-seq dataset. The method employs a gap k-mer approach and a robust method for estimating the k-mer ratio instead of a simple k-mer.

An outstanding research area in machine learning is deep learning. In recent years, deep learning has made remarkable achievements in a variety of areas such as image, natural language processing, etc. It has also enabled to draw the attention of the field of biology genetics and gradually applied in the analysis of genetic sequences. In the field of deep learning, one of the most promising neural network models is the convolutional neural network (CNN) [14]. Because of this, CNN has been introduced to the analysis of DNA sequences. As one of the earliest works to apply CNN models to sequence analysis, DeepBind [15] creatively transforms DNA sequences into 4-channel data and employs one-hot method for data processing, which allows sequence data to be effectively analyzed and predicted in CNN. Zeng was inspired by research of DeepBind to investigate the effect of different convolutional kernels and layers on sequence analysis [16]. DeepSEA [17], another classical CNN-based deep learning sequence analysis model, has expanded the number of convolutional kernels and increased the number of layers of model convolutional operations. This results in the higher-level convolutional layers to receive a larger spatial range, while the lower-level convolutional layers can capture the hidden features and perform high-dimensional representation. The model consists of three convolutional layers and two pooling layers to form the feature capture layer. The convolutional layer of DeepSNR [18] inherits the design of DeepBind [15]. The design of DeepSNR is characterized by a deconvolution operation in order to reduce the size of the activation function. Gupta [19] utilized dilated convolution to extend the range of perception of the input by the convolution operation, keeping as much information as possible without loss between the preceding and following layers. After the successful application of CNN to sequence analysis, the model design gradually started to lean toward adding new functional units, such as adding another more successful network, recurrent neural network (RNN)[20], in order to further improve the analysis. DanQ [21] added LSTM [22] after the convolutional layer to capture the long-range dependencies to improve the analysis. DeepTF used multi-nucletode one-hot (MNOH) together with multi-scale convolution and LSTM to analyze the transcription factor binding sites [23]. SAResNet analyzed transcription factor by combining residual networks(ResNet)[24] with self-attention mechanisms to form a complex deep neural network [25]. D-SCC utilized DNA sequences and shapes data and multiple deep learning modules in the model to analyze transcription factor binding sites [26]. DeepSite used the CNN method and LSTM method to capture the hidden feature of the DNA sequences [27]. However, increasingly complex models increase the number of parameters in the model. Denil [28] stated the presence of excessive parameter redundancy in deep learning models, with many parameters remaining unutilized during the training process. This redundancy leads to an unnecessary increase in the number of model parameters, resulting in issues such as heightened model complexity and increased storage requirements. Current researches do not pay attention to these issues and only pursue whether the model effect has been improved while ignoring the increase of a large number of invalid parameters. At the same time, it is undesirable to simply add an RNN model (LSTM, GRU) without considering that it will suffer from gradient disappearance and inefficiency in processing past states. In the field of DNA promoter research, Ali Raza [29] introduced iPro-TCN, a deep learning method that effectively screened promoters using Temporal Convolutional Networks (TCNs). These methods, whether they utilize TCNs or RNNs such as LSTM, have paid little attention to updating high-dimensional feature extraction methods. In this paper, it is preferred to focus on further enhancement on the formation and extraction of hidden features at each layer.

To address the aforementioned concerns, it is proposed a novel deep learning network called the Deep Concatenate Attention Augmented Convolution (DeepCAC). The method employs a multi-unit attention mechanism with a convolutional module in the feature extraction layer to form high-dimensional features, which, to the best of our knowledge, is the first time such an approach has been used in work on DNA transcription factors and has achieved good performance results in experiments comparing it with other methods. We declare the following contributions of our method: (1) We have successfully combined the self-attention mechanism with convolution and applied it to DNA transcription factors data. (2) Our method enables the simultaneous capture of local hidden features and long-distant dependent hidden features in the analysis of DNA transcription factor sequence data. (3) In comparison with other methods, our method achieves optimal results even with a small number of parameters, achieving the goal of controlling the number of parameters and improving the analysis.

## Related work

### Convolutional neural network

Modern computer vision research is built on massive image datasets containing image features. The deep learning network model of choice for learning to classify these images is the CNN. In the CNN design, the neurons in the layer responsible for extracting features are not connected to all the neurons in the adjacent layers; instead, they are connected only to the fixed-size or partially overlapping neurons of the spatial mapping of the input feature images in the previous layer. Supported by a large number of computer vision datasets, the backbone of CNN has been updated time and again to make CNN evolve and become the mainstream model in deep learning [30]. In view of the promising analytical performance of CNN, it is gradually applied to bioinformatics research and has become a research hotspot. Especially, its promising performance in the face of high-dimensional data has made it gradually show its leading role in the field, such as motif mining [31].

### Attention mechanism

With the continuous development of technology, attention, as a new deep learning computational method, has been widely used due to its ability to effectively capture long-range interaction relationship. The most noteworthy thing is that when the attention mechanism was proposed by Bahdanau et al. [32], it was mainly used in conjunction with RNN applications in the field of machine translation. It was initially designed to cope with the fact that the use of fixed-length vectors in machine translation could lead to missing information and the inability to model the alignment relationship between input and output sequences. The attention mechanism was subsequently extended by Vaswani et al. [33]. A deep learning model Transformer based on a multi-head self-attention mechanism was proposed. This model differs from any previous models in that it does not need to resort to the structure of CNN or RNN, instead relies solely on the attention mechanism for hidden feature capture. This self-attention mechanism is often proposed to be used in conjunction with CNNs in natural language processing. For example, it is used in Question Answering applications [34]. The evolving development of attention mechanisms in the field of natural language processing has also stimulated the development of research on attention mechanisms in the computer vision. Although the research on attention mechanism represented by Transformer has made some achievements, CNN has been the mainstay in machine vision. Attention mechanisms are mainly coupled with convolutional neural networks, such as BAM and CBAM [35, 36]. CNN-based models are still more susceptible to better results.

Similar to computer vision, CNN is currently the main research method in bioinformatics. Therefore, this experiment is also based on CNN. However, it is different from the previous experiments where the attention mechanism is simply incorporated into the model as an analysis module. Instead, it is  added as a computational module of multi-head self-attention mechanism for each convolution operation to form an attention-enhanced convolutional computation method. Such computational methods are concatenated in each layer to capture hidden features. Eventually, the feature maps obtained in this way enter the classifier for classification analysis.

## Method

### Overview of our method

**Fig. 1:**
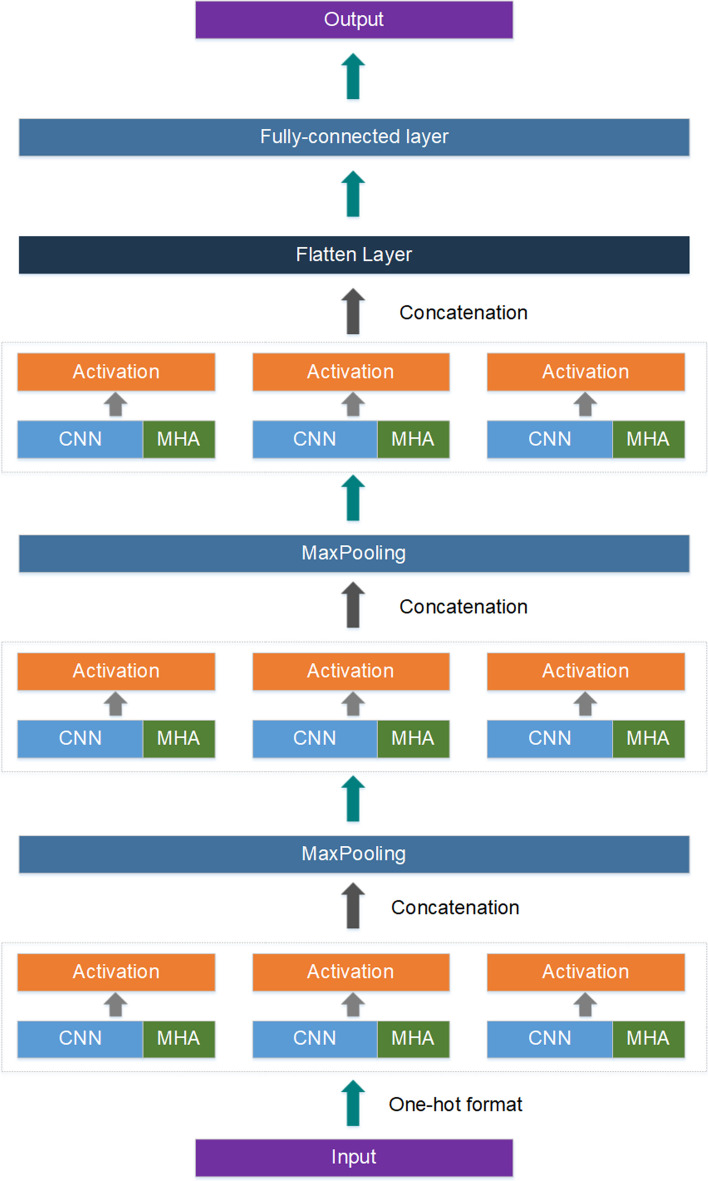
The structure of the model

We developed a concatenate deep learning model based on convolution operation and multi-head self-attention mechanism, which is named DeepCAC that can automatically capture and learn heterogeneous hidden features in DNA sequences. As shown in Fig. 1, each layer in the model contains two main modules, one is a convolution module and the other is a multi-headed self-attention module. The organization of these two modules is mainly based on the attention augmented convolutional module [37], which form a complete feature vector by concatenating the feature vector of convolution and the feature vector of multi-head self-attention.

Expressly, in this experiment, the overall network structure is composed of three concatenate attention augmented convolution layers, two pooling layers, and two fully connected layers. Each layer in the whole network is composed of three parallel concatenate attention augmented convolutional modules and three activation functions. This network layer scans the DNA sequences and captures the hidden features present in their sequences. Eventually, the high-level feature vector after multiple feature extractions will be output to the two-layer fully connected layer, which will be used as a predictor to classify and predict the relevant attributes of the sequence for analysis. The following formula needs to be satisfied in the learning process

1 $$\upzeta =-\sum\nolimits_{i=1}^{n}{y}_{i}\sigma (\alpha \cdot {H}_{i}+\beta )+(1-{y}_{i}\mathrm{log}(1-\sigma (\alpha \cdot {H}_{i}+\beta )))$$

In the formula, both $\alpha$ and $\beta$ are parameters of the output of the method after learning the data. ${y}_{i}$ is the true label.$\sigma$ is the activation function.${H}_{i}$ is the input feature vector. The training process is to obtain the $\alpha$ and $\beta$ that make the minimum $\upzeta$. The overall process is shown in Algorithm 1.

**Algorithm 1:**
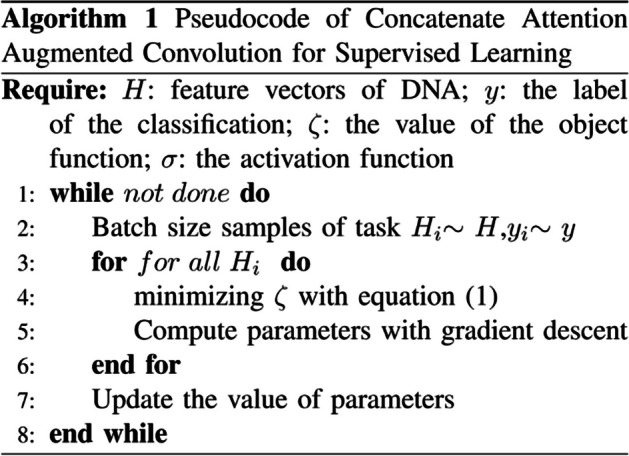
Pseudcode of DeepCAC for Supervised Learning

### Multi-head self-attention mechanism

In this study, for a given input feature vector $x$, its shape is $[L, D]$, where $L$ is the length of the feature vector extracted from the sequence, and $D$ is the dimension of the feature vector input to the multi-head self-attention mechanism. Here, we set the whole feature $X$ as a matrix $X=[{x}_{1}, { x}_{2}, {x}_{3},\dots , {x}_{n}]$. In the system of self-attention mechanisms, there are three very important feature vectors, $Q$, $K$, and $V$, and we set $Q= K= V= X{W}_{i}$, where ${W}_{i} \in [{W}_{Q}, {W}_{K}, {W}_{V}]$. ${W}_{i}$ is the parameter that the model learns from training. The self-attention mechanism, as a method that can focus on global features, calculates the weights of each feature vector in the overall feature. These weights reflect those feature vectors that play a more critical role for the model. These weights are then assigned to each feature vector to form the new overall feature vector output from the module. This process satisfies the following formula:

2 $$Head(X)=Softmax\left(\frac{\left(X{W}_{Q}{\left(X{W}_{k}\right)}^{T}\right)}{\sqrt{{d}_{k}}}\right)(X{W}_{V})$$

In the formula, ${d}_{k}$ is the dimension of $K$, which is used in the formula to reduce the computational complexity. The output of this part is the content of one head. In this experiment, a multi-head self-attention mechanism (MHA) is applied, in which the output of each head is integrated by a unified concatenate method after the output of each head. The method satisfies the following formula:

3 $${Output}_{X}=Concat[{Head}_{1}\left(X\right), {Head}_{2}\left(X\right), {Head}_{3}\left(X\right)\dots , {Head}_{n}\left(X\right)]{W}^{o}$$

${W}^{o}$ is a learned linear transformation. After this, there will be a reshape method to reshape the output vector into the target dimension. The formula is:

4 $$MHA\left(X\right)=Reshape({Output}_{X})$$

### Concatenate attention augmented convolution module

As mentioned above, attention augmented convolution was used in this experiment. In this method, the output dimension is concatenated from the dimension of the convolution operation and the output dimension of the MHA mechanism. It satisfies the following formula:

5 $${Output}_{Conv\_MHA}(X)= Concat[Conv\left(X\right), MHA(X)]$$

Here, the output dimension is set to ${d}_{out}$, which is the output dimension of ${Output}_{Conv\_MHA}(X)$. On the right side of the formula, the output of the MHA is the vector that the dimension of the vector $V$ is set as ${d}_{V}$. Therefore, the output dimension of the convolution part is ${d}_{Conv}= {d}_{out}- {d}_{V}$, and ${d}_{Conv}$ is the number of filters in the convolution part.

In the overall experiment, the sequence contains a variety of hidden features and they are not easily detected. Therefore, it is conjectured that a module composed of multiple attention augmented convolution is more helpful for the model to discover different potential hidden features. Based on such conjecture, the concatenate attention augmented convolution module is designed to capture the sequence hidden features. It satisfies the following formula:

6 $$H(X) \,=\, Concat\left[\sigma \left({{Output}_{Conv\_MHA}\left(X\right)}_{1}\right), \sigma \left({{Output}_{Conv\_MHA}\left(X\right)}_{2}\right),\dots , \sigma \left({{Output}_{Conv\_MHA}\left(X\right)}_{i}\right)\right]$$

In this formula, the $\sigma$ presents the activation function and $i$ is the number of the ${Output}_{Conv\_MHA}$. $H(X)$ is the high-dimensional feature vectors of the DNA sequence. Here, the output dimensions were also divided, which was divided equally, that is, ${d}_{Output}=\frac{ D}{i}$. $D$ is the dimension of the $H(X)$ and ${d}_{Output}$ is for the dimension of the each ${Output}_{Conv\_MHA}$. It is used as a layer in conjunction with other methods to extract the features in the sequence. The overall algorithm process is shown in Algorithm 2.

**Algorithm 2:**
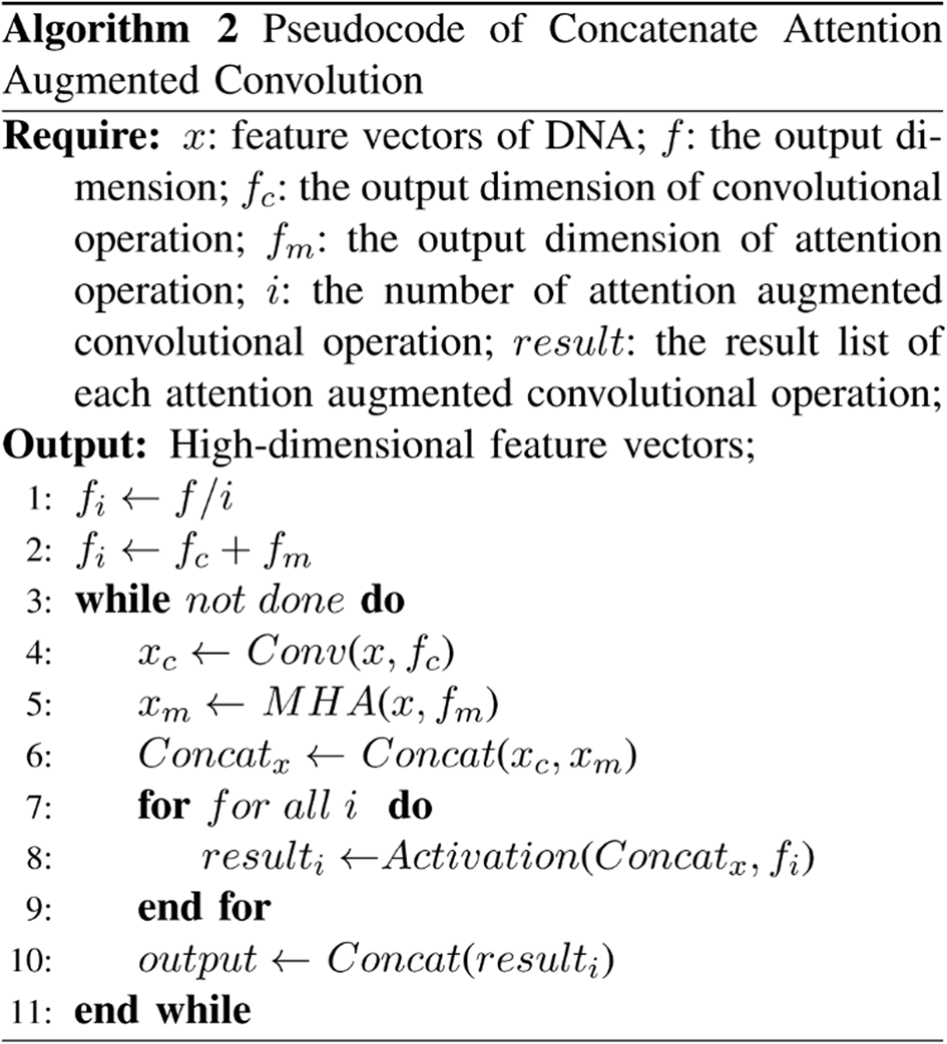
Pseudcode of Concatenate Attention Augmented Convolution in DeepCAC

## Experiment

### Data source

In the experimental design for DNA transcription factors prediction, DNA transcription factors data produced by Koo et al. [38] was utilized. This dataset contains ENCODE ChIP-seq peak results. Chromosomes 8 and 9 were used as the test set, while the rest of the chromosome data was used for training and validation. In Koo's data design, 12 transcription factors, Arid3, CEBPB, FOSL1, Gabpa, MEF2A, MAFK, MAX, MEF2A, NFYB, SP1, SRF, STAT1 and YY1, were selected to construct the dataset. 92% of the data was allocated to the training set while the remaining 8% was used as the validation set during the training process. The data was constructed in 4 × 1000 one-hot binary matrices, corresponding to A, C, G and T in every 1000nt DNA sequence.

### Experiment settings

Our method was built with Keras in python, which uses Tensorflow as the backend. For the models of experimental and different control groups in the experiment, the choice of optimizer was SGD in all methods and accelerated by GPU. A dropout method is applied to the model in order to suppress the effects of overfitting. The ratio of dropout is 0.2 in the feature extraction layer and 0.5 in the fully connected layer. The Early-stopping method was used in the experiment, and the maximum training epoch was 200. Besides, a checkpoint was designed to monitor the performance of the correctness of the validation set. When the accuracy of validation continues to decrease until 20 epochs are reached, training is stopped and the model with the highest accuracy in the training results is saved. In the overall experiment, the size of the max pooling layer was 4 and the step was also 4.

### Evaluation

The results of the experiments are discussed in terms of top-k accuracy, area under receiver operating characteristic (ROC) curve (AUC) and average precision score (AP) considering that the experiment on transcription factors is a multiclassification problem. Top-k accuracy is used to calculate the sum of the probabilities of the top k predictions. When presenting the effect on each class, precision recall curve (PRC) and ROC are used for the presentation. These two curves provide a good demonstration of the analysis effect of each method on different classes.

## Result

In order to evaluate DeepCAC effectively, DanQ [21], DeepSite [27], CNN-Zeng [16] and CNN-BiGRU were selected for comparison experiments. the design of CNN-Zeng model uses 128 convolutional kernels for feature extraction as reported in the paper, and this design is one of the models that obtains the best results. Both DanQ and DeepSite use LSTM with convolution for feature extraction. CNN-BiGRU is based on the design of DanQ with the final feature extraction layer changed to BiGRU. All methods are retrained and validated on this experimental dataset. The results of the training figures are shown in the Additional files 1, 2, 3, 4.

**Fig. 2:**
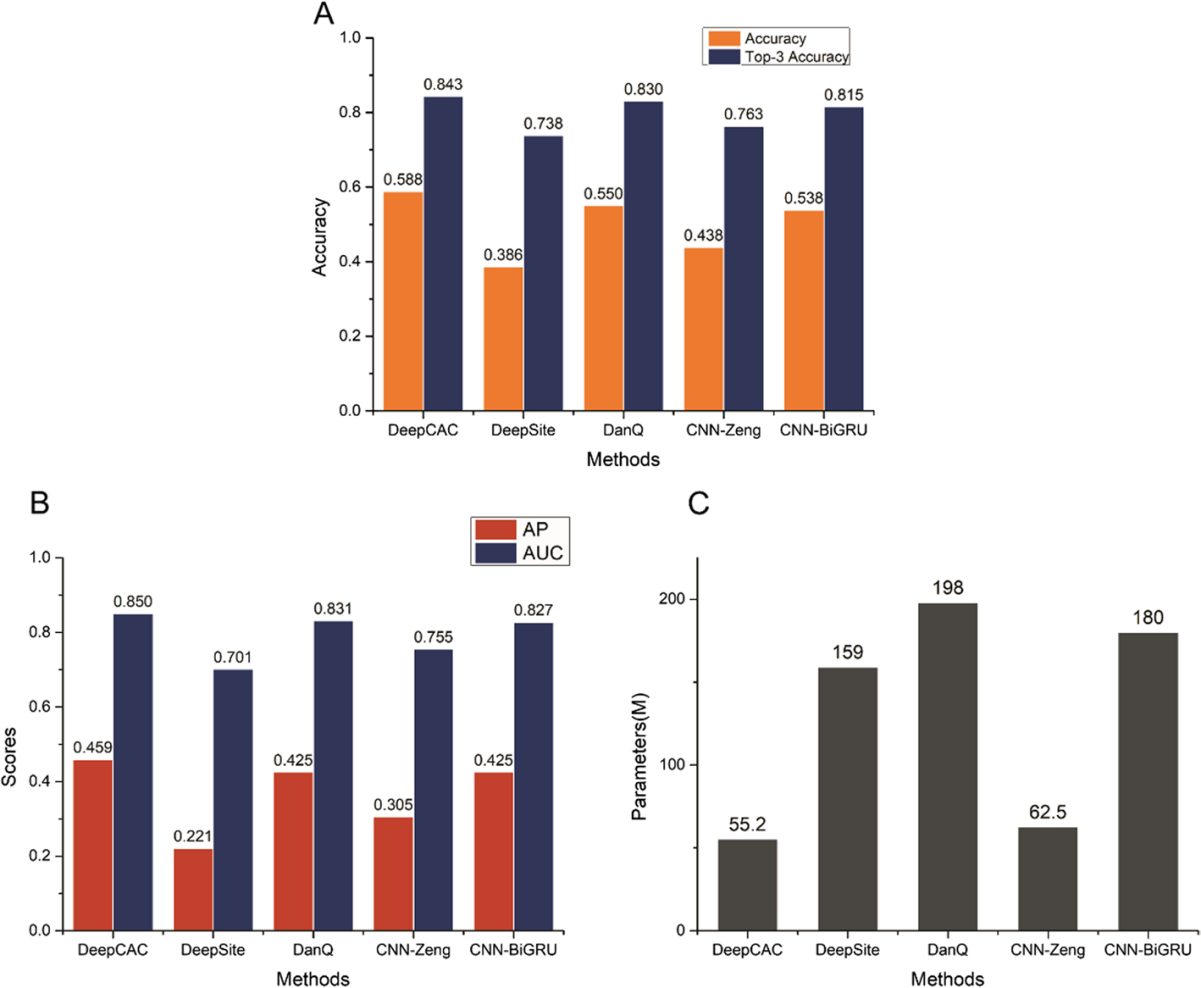
The results of the DNA transcription factors classification. **A** The accuracy performance of each methods. **B** The AUC and AP results of each methods. **C** The parameters of each methods

In this study, the model training results were evaluated using four metrics: accuracy, AUC, AP and number of parameters, and the results are shown in Fig. 2. In the accuracy comparison, DeepCAC achieved the best performance results among the five methods. In the comparison with DanQ, the accuracy was 6.9% higher, and also exceeded 1.3% in the top-3 accuracy comparison. In the comparison with CNN-BiGRU, DeepCAC also leads, with 9.2% and 3.4% higher accuracy and top-3accuracy, respectively. In the comparison of AUC, DeepCAC also showed remarkable results. It led the whole experimental results with a performance effect of 0.850. It is 1.9% higher than the second-place DanQ. DeepCAC was 2.7% higher than CNN-BiGRU. The absolute advantage is also reflected in the performance of AP. These advantages show that DeepCAC is much closer to the true value in the prediction results. And these leading effects do not rely on increasing the number of parameters to achieve. These results show that DeepCAC can achieve better analysis results without increasing the number of parameters.

To demonstrate the analytical effect on different classes, the AUC result of each class are listed in Table 1 for comparison. Overall, DeepCAC achieved a more significant lead in 10 of the 12 classes, and did not achieve good analytical results only in SP1 and SRF. Among these leading 10 classes, DeepCAC obtained the most obvious results on FOSL1, with a 5.8% improvement in AUC score compared to CNN-BiGRU. DeepCAC's AUC peaked in the NFYB class at 0.950. In the peak comparison, DanQ and CNN-BiGRU also achieved peaks of 0.926 and 0.919 in the NFYB class, while CNN-Zeng had the best performance in the YY1 class at 0.858. DeepSite, like CNN-Zeng, also achieves a peak of 0.845 in YY1. Among these peaks, DeepCAC also leads the other four methods by a minimum margin of 2.6%. The class with the smallest lead was YY1, on which the five methods achieved almost close performance results, with a maximum gap of 4% ahead of DeepSite. In the two classes where DeepCAC performed poorly, the largest gap appeared in SRF, with a gap of 1.8%. In these two classes of poor performance, the best results were achieved by DanQ and CNN-BiGRU, respectively. The possible reason for this situation is that DeepCAC is still not obvious enough in capturing the long-range dependency in the feature vector to analyze the information contained in the longer-range features.

**Table 1:** The AUC result for each class

TFs	DeepCAC	DanQ	DeepSite	CNN-Zeng	CNN-BiGRU
Arid3a	**0.808**	0.785	0.678	0.699	0.781
CEBPB	**0.940**	0.896	0.706	0.774	0.886
FOSL1	**0.909**	0.805	0.645	0.760	0.851
Gabpa	**0.832**	0.826	0.776	0.788	0.818
MAFK	**0.925**	0.891	0.721	0.773	0.886
MAX	**0.848**	0.835	0.784	0.805	0.813
MEF2A	**0.735**	0.712	0.555	0.622	0.723
NFYB	**0.950**	0.926	0.680	0.842	0.919
SP1	0.797	**0.808**	0.765	0.780	0.792
SRF	0.774	0.790	0.665	0.706	**0.792**
STAT1	**0.790**	0.776	0.594	0.667	0.785
YY1	**0.885**	0.884	0.845	0.858	0.879

The best result is marked in bold font

**Fig. 3:**
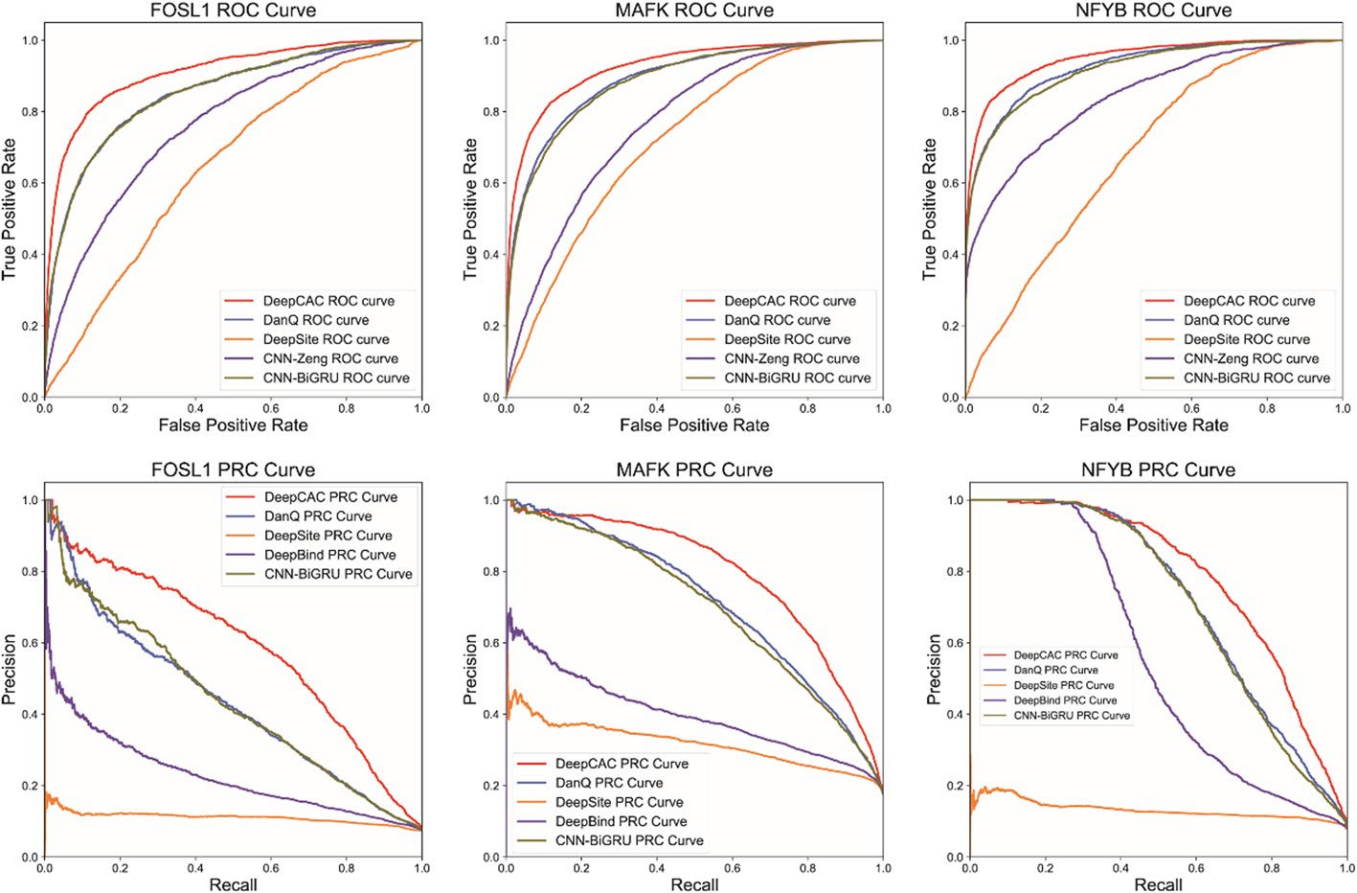
The ROC and PRC curve of FOSL1, MAFK and NFYB

For better demonstration of the analysis, Fig. 3 shows the ROC and PRC figures for some of the classes. In these three classes shown, the curves of DeepCAC were significantly higher than those of the other four methods. The advantage was also maintained in the CNN-BiGRU with DanQ that contains RNNs. With the information in figures, it can be seen that adding the analysis module did improve the analysis to some extent, but not all models achieved better and desirable results. In terms of overall performance, DeepSite did not perform very well on these 12 types of datasets, probably because its design approach did not capture effective hidden features. The full ROC and PRC figures are provided in the Additional files 1, 2, 3, 4.

Experiments were executed on the effect of different number of heads on the training time in the multi-head self-attention mechanism. To expedite the validation process, the original data length was reduced from 1000nt to 100nt in this experiment. To compare their effects, 8, 16, 20 and 30 heads were tested and the observed one epoch times were 1170.07, 1949.33, 2357.94 and 3870.07 s, respectively. These results indicate that an excessive number of heads slows down the training process and decreases efficiency. Considering DeepCAC’s aim to enhance experimental efficiency without compromising overall performance, 8 heads were selected as the optimal choice. Subsequent experimental results confirmed that employing 8 heads was sufficient to achieve effective outcomes. The impact of different numbers of heads on the experiment is depicted in the Additional files 1, 2, 3, 4.

## Discussion

Gene expression and regulation involve numerous processes, and transcription, as the first step of gene expression, is central to the regulatory mechanism of gene expression. The process of transcription initiation in eukaryotes is complex and transcription factors are key components. First, universal transcription factors participate in the general transcription process to enable the initiation of gene expression. Second, tissue- and cell-specific transcription factors are required for expressing specific protein molecules in particular tissue cells at different times. Therefore, accurately identifying target transcription factors is essential for studying both gene expression regulation and biological processes. Using computational methods to rapidly identify transcription factors has gradually become an important requirement in bioinformatics. The transcription factor classification designed here is based on convolution and incorporates a multi-headed self-attention mechanism, concatenating multiple such convolution operations. This method captures hidden features in the sequence, learns them, and provides feature vectors to the classifier for prediction. It can effectively transform the captured hidden features into a high-dimensional feature vector for the classifier. To evaluate the method's validity, relevant experiments were designed for analysis and verification. The experiments show DeepCAC is practical and effective.

Compared to the other four methods, the high-dimensional feature vector generated by DeepCAC may contain richer features. Unlike general deep learning methods, the multi-head self-attention mechanism can capture long-distance dependencies in the computation. By fusing regular convolution operations and the multi-head self-attention mechanism, the number of parameters is reduced after model generation. As Geiger [39] emphasizes, while deep learning methods have achieved remarkable performance across various fields, many of these achievements are based on overfitting a large number of parameters. Therefore, this paper aims to propose a deep learning model with a more reasonable number of parameters to achieve accurate prediction of DNA transcription factors. Meanwhile, convolutional computation is retained to efficiently capture local hidden features. Concatenating multiple self-attention augmented convolution operations also enables the method to effectively handle different sequences. Based on its optimal performance on the DNA transcription factor task, this method could be applied by other researchers to construct neural network models for various analysis targets.

This experiment seeks to update the convolution itself to achieve a reduced dependence on the number of parameters. Compared with the SAResNet [25] and D-SCC models [26], this experiment focuses more on updating the methods in each layer of the model to further improve the data analysis, rather than stacking deep learning analysis modules for simply pursuing results. In our future research, it will be used as the basic analysis unit and combined with ResNet [24] and DenseNet [40] to further improve the analysis capability while further controlling the number of model parameters within a certain range. DeepCAC is not designed to apply the Transformer model directly as DNABERT [41] does. Using the Transformer model requires the slicing method of sequences, which would have a problem of uncertainty slicing length of sequences. The principles underlying DeepCAC have the potential to be applied in other domains, such as proteins and tuberculosis research. For instance, in studies involving ensemble models like iHBP-DeepPSSM [42] and iAtbP-Hyb-EnC [43], DeepCAC could serve as a valuable component. Additionally, DeepCAC is likely to exhibit effectiveness in disease and drug discovery domains, including miRNA-disease [44], RNA 5-methylcytosine [45] and drug repositioning [46]. These areas present crucial avenues for future research.

## Conclusion

This paper proposes a concatenated attention augmented convolution layer design and applies it to analyze DNA transcription factor sequences, efficiently capturing hidden features with a reasonable number of parameters. This method is called DeepCAC. In previous studies on DNA transcription factor sequences, there has been limited focus on designing each layer of deep learning methods, leading to overemphasis on increasing model complexity to improve analytical capability. Extensive experimental data shows that DeepCAC can achieve state-of-the-art performance compared to classical convolutional methods. With appropriate hyperparameter tuning for different characteristic gene sequences, optimal results can be achieved overall. Future work will further expand the DeepCAC concept to design more complex modeling frameworks. The research will also explore areas beyond transcription factors, such as diseases and drug discovery. In summary, this research on improving convolution aims to provide new perspectives and analytical ideas for analyzing DNA transcription sequences with deep learning. It also hopes to bring different experimental design approaches to the broader field combining bioinformatics and deep learning.

### Supplementary Information

**Additional file 1: Fig. S1**. The ROC curve for each class.

**Additional file 2: Fig. S2.** The PRC curve for each class.

**Additional file 3: Fig. S3**. The training figure of each method.

**Additional file 4: Fig. S4**. Impact of different number of heads on the runtime of a single epoch.

## Data Availability

The dataset of DNA transcription factors classification can be downloaded from https://github.com/p-koo/learning_sequence_motifs/tree/master/code. The code used or analyzed during this study are available from the corresponding author on reasonable requests.
