# Supplementary material for: DeepCAC: a deep learning approach on DNA transcription factors classification based on multi-head self-attention and concatenate convolutional neural network
Source: BMC Bioinformatics. 2023 Sep 18;24:345. doi: 10.1186/s12859-023-05469-9 (PMC10506269; doi:10.1186/s12859-023-05469-9)
Supplement: Supplementary file 2 — Additional file 2: Fig. S2. The PRC curve for each class. [file 12859_2023_5469_MOESM2_ESM.pdf]

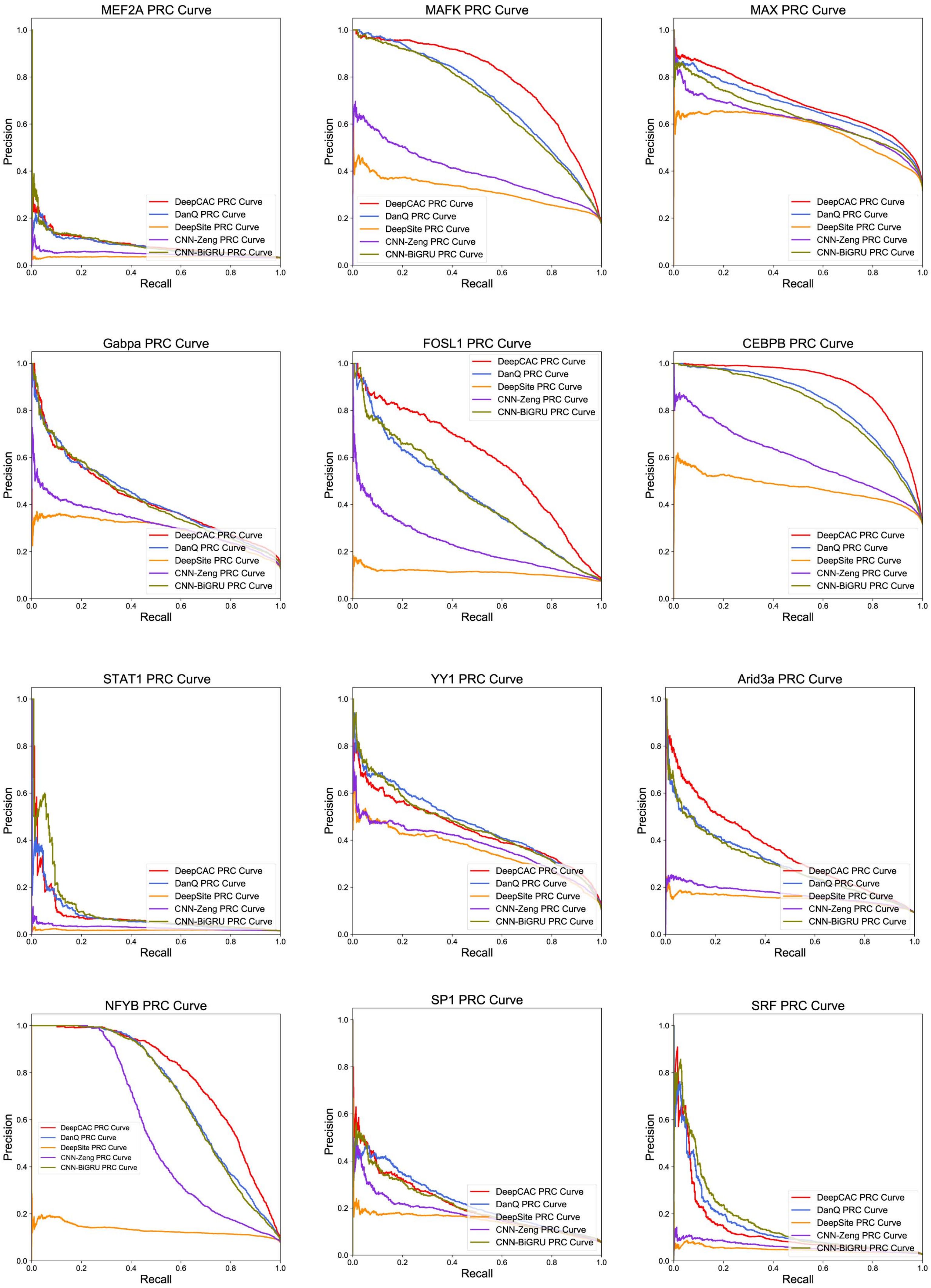
MEF2A PRC Curve
MAFK PRC Curve
MAX PRC Curve
Gabpa PRC Curve
FOSL1 PRC Curve
CEBPB PRC Curve
STAT1 PRC Curve
YY1 PRC Curve
Arid3a PRC Curve
NFYB PRC Curve
SP1 PRC Curve
SRF PRC Curve
Precision
Recall
DeepCAC PRC Curve
DanQ PRC Curve
DeepSite PRC Curve
CNN-Zeng PRC Curve
CNN-BiGRU PRC Curve
