# Supplementary material for: DeepCAC: a deep learning approach on DNA transcription factors classification based on multi-head self-attention and concatenate convolutional neural network
Source: BMC Bioinformatics. 2023 Sep 18;24:345. doi: 10.1186/s12859-023-05469-9 (PMC10506269; doi:10.1186/s12859-023-05469-9)
Supplement: Supplementary file 3 — Additional file 3: Fig. S3. The training figure of each method. [file 12859_2023_5469_MOESM3_ESM.pdf]

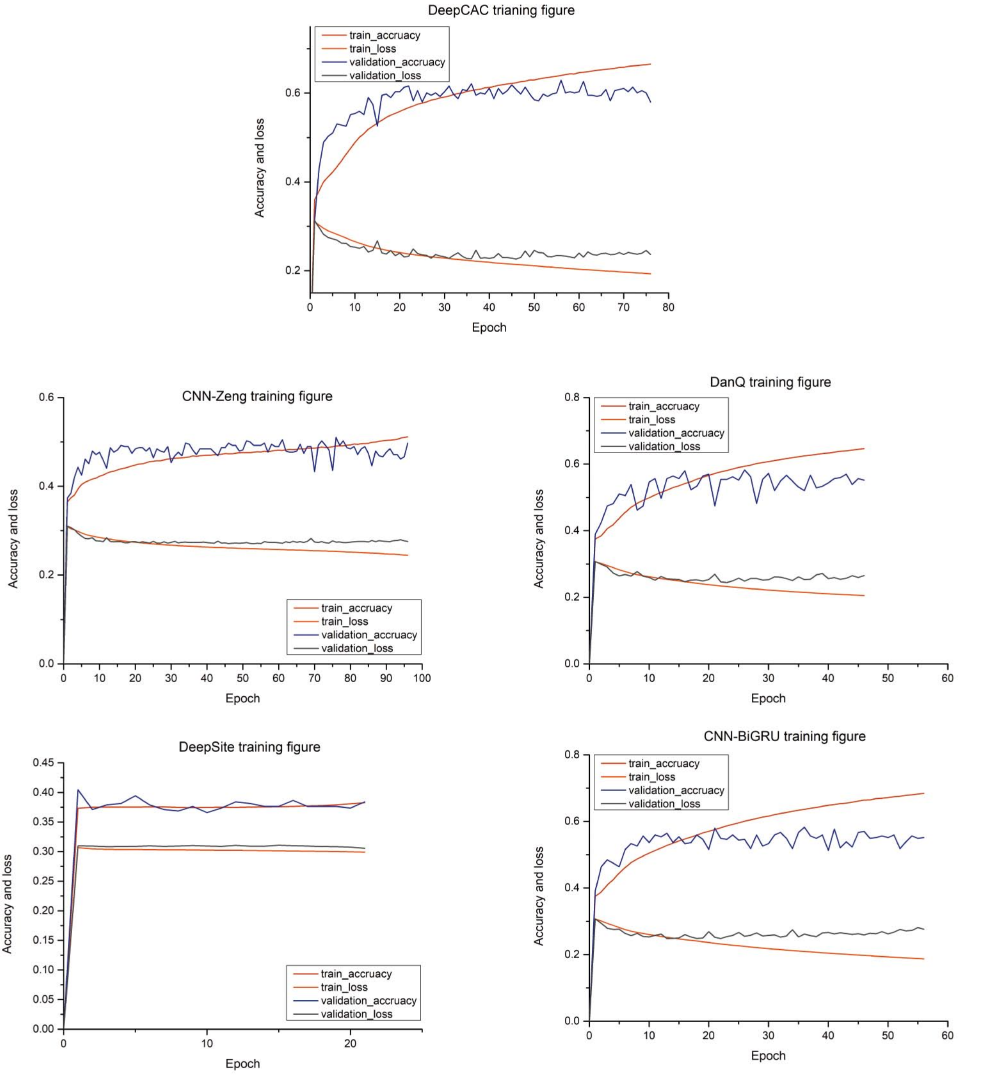
DeepCAC trianing figure
train_accruacy
train_loss
validation_accruacy
validation_loss
Accuracy and loss
Epoch
0.2
0.4
0.6
0
10
20
30
40
50
60
70
80
CNN-Zeng training figure
Accuracy and loss
Epoch
0.0
0.2
0.4
0.6
0
10
20
30
40
50
60
70
80
90
100
train_accruacy
train_loss
validation_accruacy
validation_loss
DanQ training figure
train_accruacy
train_loss
validation_accruacy
validation_loss
Accuracy and loss
Epoch
0.0
0.2
0.4
0.6
0.8
0
10
20
30
40
50
60
DeepSite training figure
Accuracy and loss
Epoch
0.00
0.05
0.10
0.15
0.20
0.25
0.30
0.35
0.40
0.45
0
10
20
train_accruacy
train_loss
validation_accruacy
validation_loss
CNN-BiGRU training figure
train_accruacy
train_loss
validation_accruacy
validation_loss
Accuracy and loss
Epoch
0.0
0.2
0.4
0.6
0.8
0
10
20
30
40
50
60
