# Supplementary figures and images for: DeepCAC: a deep learning approach on DNA transcription factors classification based on multi-head self-attention and concatenate convolutional neural network
Source: BMC Bioinformatics. 2023 Sep 18;24:345. doi: 10.1186/s12859-023-05469-9 (PMC10506269; doi:10.1186/s12859-023-05469-9)

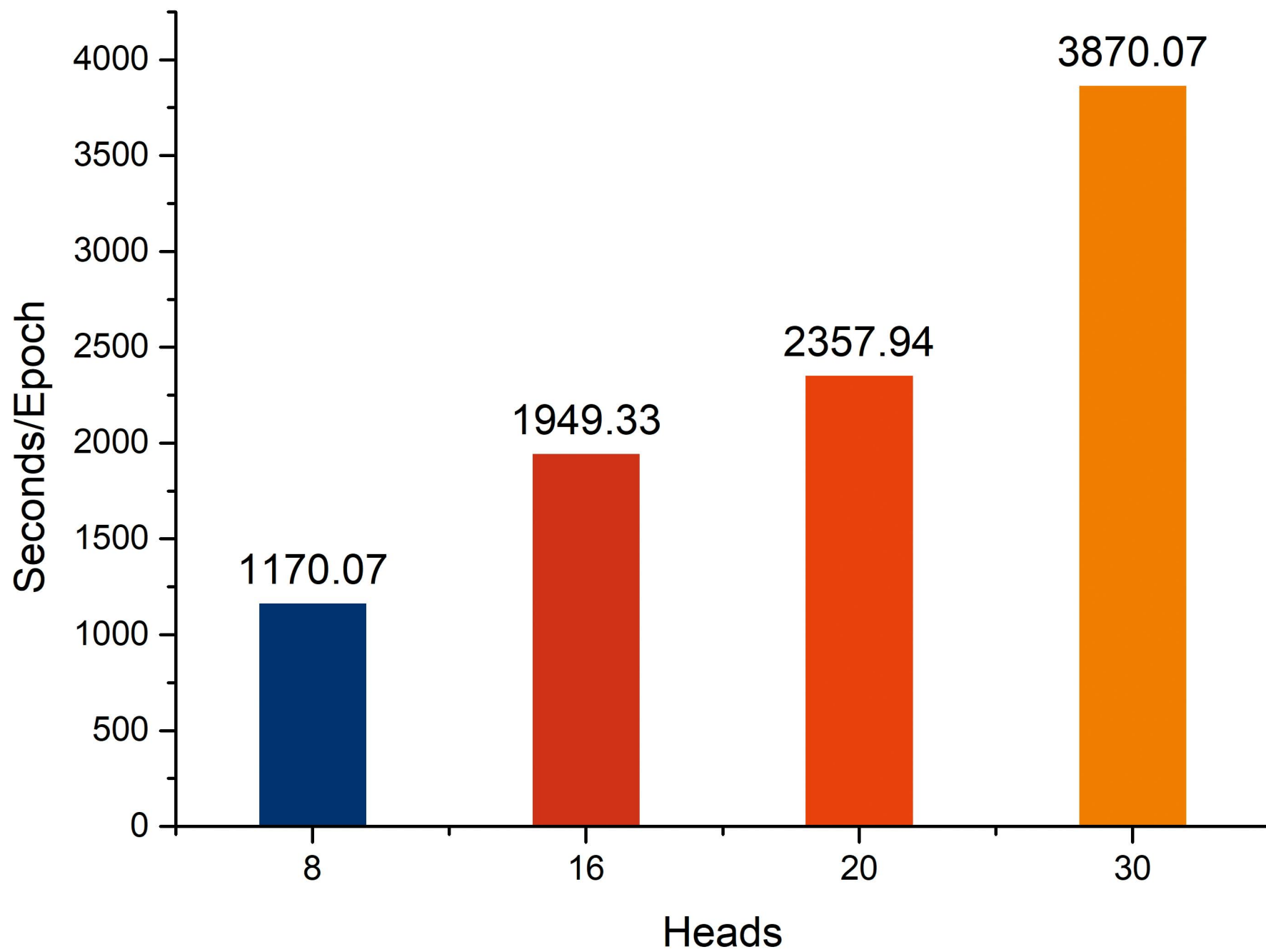

Supplement: Supplementary file 4 — Additional file 4: Fig. S4. Impact of different number of heads on the runtime of a single epoch. [file 12859_2023_5469_MOESM4_ESM.pdf]
