# Supplementary material for: DeepCAC: a deep learning approach on DNA transcription factors classification based on multi-head self-attention and concatenate convolutional neural network
Source: BMC Bioinformatics. 2023 Sep 18;24:345. doi: 10.1186/s12859-023-05469-9 (PMC10506269; doi:10.1186/s12859-023-05469-9)
Supplement: Supplementary file 1 — Additional file 1: Fig. S1. The ROC curve for each class. [file 12859_2023_5469_MOESM1_ESM.pdf]

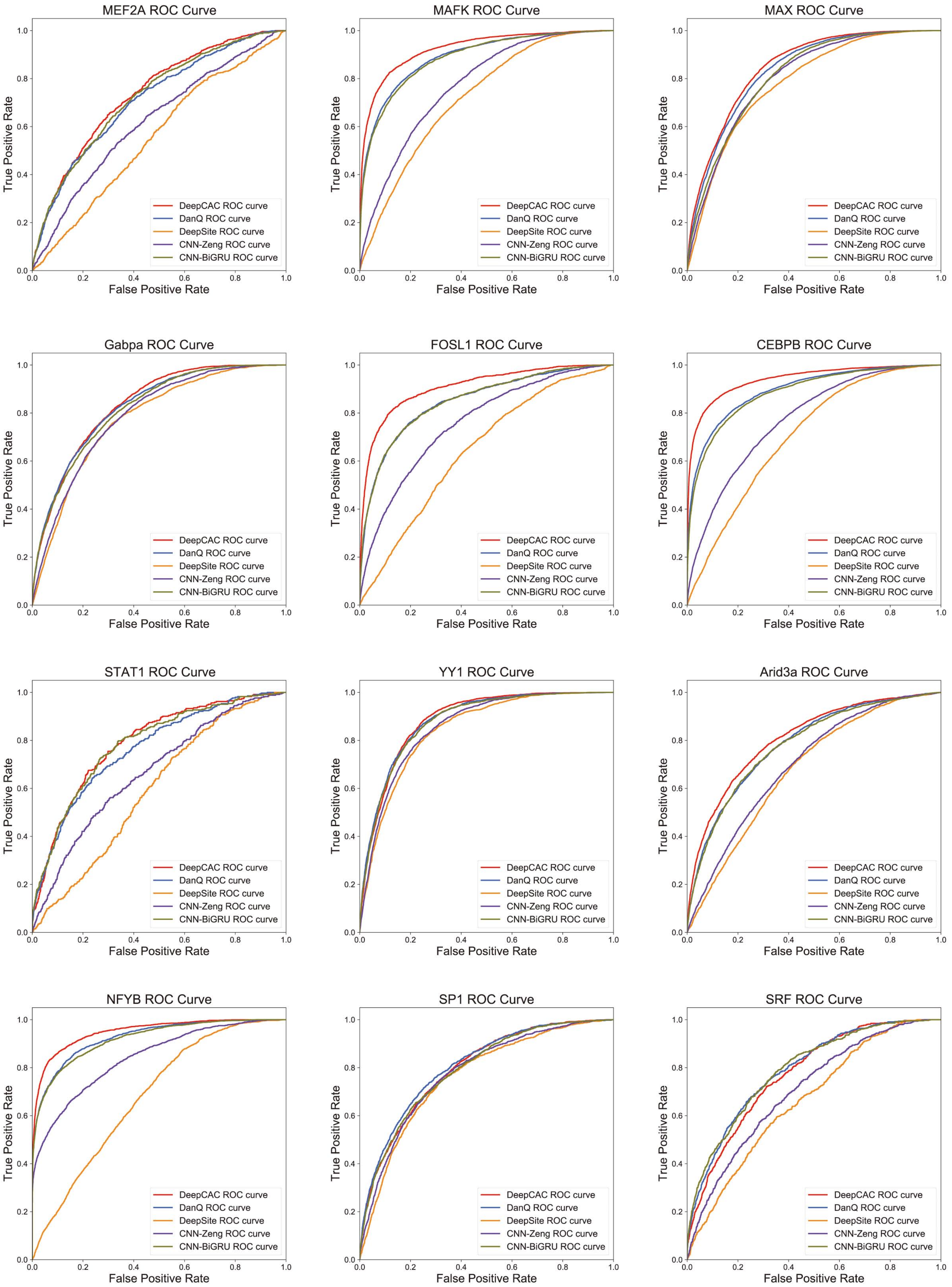
MEF2A ROC Curve
MAFK ROC Curve
MAX ROC Curve
Gabpa ROC Curve
FOSL1 ROC Curve
CEBPB ROC Curve
STAT1 ROC Curve
YY1 ROC Curve
Arid3a ROC Curve
NFYB ROC Curve
SP1 ROC Curve
SRF ROC Curve
True Positive Rate
False Positive Rate
0.0
0.2
0.4
0.6
0.8
1.0
DeepCAC ROC curve
DanQ ROC curve
DeepSite ROC curve
CNN-Zeng ROC curve
CNN-BiGRU ROC curve
